# Western Texas as a Health Resort

**Published:** 1881-01

**Authors:** 

**Affiliations:** San Antonio, Texas


### WESTERN TEXAS AS A HEALTH RESORT.

San Antonio, Texas, January 1, 1881.

Editor of Hall's Journal of Health :

Dear Sir : There are few regions anywhere, no matter what their condition as to healthfulness or the reverse may be, the inhabitants of which are willing to admit that they are unsafe. There may be *malaria* in the neighborhood, but it is always a little way removed from the place inquired about. There are thousands of places in the new States where fever and ague invariably attack new-comers, which are described by railroad and land agents as being perfectly healthy. There is more or less *malaria* in nearly all new countries. and many old and long-settled regions are not free from it. A most valuable and useful paper would be a faithful report from every practicing physician in every State, giving a list of the diseases treated by them in the course of a year's practice. I fear that chills and fever and kindred diseases would figure more largely in such a statement than most persons are prepared to expect. As to Texas, I find after careful inquiry that in nearly all the Eastern or timber countries and along nearly all the river bottoms *malaria* exists, but away from these and in nearly the whole of Central and of Western Texas it is practically unknown. One may go farther than this, and say that there is an extensive and beautiful region in Western Texas, not only exempt from any danger of chills and fever, but where pulmonary diseases are arrested and often cured. A large section of the State, not far from the quaint and pretty city of San Antonio, offers a safe and desirable resort to those threatened with or suffering from consumption and kindred diseases. This region is elevated from 800 to 1,200 feet above the sea, and is perpetually fanned by the breezes from the Gulf of Mexico. The air is exceptionally dry and bracing, the Winter temperature rarely falls so low as the freezing point, that of Summer never reaching the temperature of St. Louis and many other Northern towns. The present Winter has, thus far, been an exceptionally cold one here, as it has been everywhere else, and the cold has been more intense and prolonged than ever before known by persons now living ; but the atmosphere has been always clear and dry, and the invalids who are known to me assure me that they have suffered no inconvenience from the cold.

It has long been claimed by those interested in this region of Texas, that pulmonary disease is almost always arrested and very frequently cured by a prolonged residence in the region round about San Antonio. I have taken some pains to verify this statement, and am compelled to admit that the claim is well founded. One meets here with many persons who state that they were considered hopelessly ill when they left the North or Europe, and that they are now well. There are several well-known and reputable business men in San Antonio whom I have met, who assure me that they literally came here to die. They are now to all appearance well. A prominent citizen, who was once a resident of Chicago, assures me that when he came here, a dozen years ago, he was a confirmed consumptive, and the lite insurance company in which his life had been insured was glad to compromise with him by the payment of a large sum of money. He accepted it, came here and is now quite well. I have no reason to doubt the statement, which is every day made, that there are hundreds of like cases. I may sum up the whole matter by stating that I am fully of the opinion that there is no part of the United States the climate of which is so well adapted to the alleviation and cure of pulmonary disease as that of the higher regions of Southwestern Texas. While as a Winter residence it is preferable to Florida or Southern California, it is an agreeable and comfortable residence for the Summer months as well.

One must be prepared to discredit the repeated statements of intelligent and reputable witnesses who denies that many cases of pronounced consumption have been cured by a lengthened residence in the region referred to. It must be admitted that the results are not always favorable, for we see here many poor sufferers who have evidently come too late.

I have no hesitation in advising persons who are suffering from diseases of the air passages to try the experiment of a residence here.

It is much to be regretted that a region so admirable and desirable as a health resort should lack to such a degree the means of making those who resort to it comfortable. With rare exceptions the hotels of Texas are abominable, and the boarding-houses are only just fearable. The average Texan seems to know nothing of good cooking, and he cares less. With cheap and excellent markets, he is contented with a cuisine that is worse than that of the poorest German village. With good cows selling at twelve dollars each, and with pasture everywhere free and perennial, he is content to purchase New York oleomargarine at a high price, and with few exceptions, he uses only New York or Swiss condensed milk in his tea and coffee. One who has been but a few days in Texas will appreciate the remark made to me by a leading railway official, who said that if all native Texans could be forced to spend a few weeks of their lives in the North, to see how northern people live, Texas would soon become the most agreeable place of residence in the world.

I hear of only a few places where an invalid would be at all comfortable, and these few I can and will name. I shall gladly add others in future letters, should I hear of them. At San Antonia Capt. Tobin has a comfortable boarding house; at Seguin, the Magnolia House is well spoken of, as is the Boerne Hotel, at Boerne.

In addition to other attractions for invalids possessed by this wonderful Etate, I may mention the mineral waters which are found in many places. As usual where mineral springs exist, the most extravagant claims are set up, and they are declared to be specifics for all known diseases. I have not been able to find a single well authenticated scientific analysis of any of the Texas mineral waters, and know nothing therefore of the value of any os them as curative agents.

I am having samples of several of the springs prepared for transmission to the *Journal of Health.* Your scientific analysis I shall be only too glad to publish for the benefit of all concerned. In a future letter I hope to be able to give some valuable information in relation to the healing springs of Texas.

Yours etc., ANGLO-SAXON.

### LET SOME OLDER ONES TRY.

Class in arithmetic, fall in! The boys fall in. Teacher—Now, boys, what is this I have in my hand? All the Boys—It's a dollar. Teacher—Yes, it's a legal-tender dollar. It is called a dollar of the fathers. How much silver does it contain? Small Boy—412½ grains. Teacher—That's right. Now, what do you call this? It is also a silver dollar, but what is it called? Small Boy (after examination)—It's a trade dollar. Teacher—That's right. Now, how much silver does it contain? Small Boy—420 grains. Teacher—How much is it worth? No answer from the boys. Teacher—Well, it is worth ninety cents. All the Boys—It is worth ninety cents. Teacher—Now, boys, tell me why it is that the dollar containing 412½ grains of silver is worth one hundred cents, while the dollar containing 420 grains is worth only ninety cents? Head of Class—Blamfino. Teacher—The class is dismissed.

